# Integrating 16S rRNA Sequencing, Microflora Metabolism, and Network Pharmacology to Investigate the Mechanism of SBL in Alleviating HDM-Induced Allergic Rhinitis

**DOI:** 10.3390/ijms25168655

**Published:** 2024-08-08

**Authors:** Peiting Li, Sharon Sze-Man Hon, Miranda Sin-Man Tsang, Lea Ling-Yu Kan, Andrea Yin-Tung Lai, Ben Chung-Lap Chan, Ping-Chung Leung, Chun-Kwok Wong

**Affiliations:** 1 Institute of Chinese Medicine, State Key Laboratory of Research on Bioactivities and Clinical Applications of Medicinal Plants, The Chinese University of Hong Kong, Hong Kong, China; peiting@link.cuhk.edu.hk (P.L.); sharonhon@link.cuhk.edu.hk (S.S.-M.H.); miranda.tsang@rmit.edu.au (M.S.-M.T.); lea-kan@cuhk.edu.hk (L.L.-Y.K.); andrealai@link.cuhk.edu.hk (A.Y.-T.L.); benchan99@cuhk.edu.hk (B.C.-L.C.); pingcleung@cuhk.edu.hk (P.-C.L.); 2 Department of Chemical Pathology, The Chinese University of Hong Kong, Prince of Wales Hospital, Hong Kong, China; 3 China-Australia International Research Centre for Chinese Medicine, School of Health and Biomedical Sciences, STEM College, RMIT University, Bundoora, VIC 3083, Australia; 4 Li Dak Sum Yip Yio Chin R&D Centre for Chinese Medicine, The Chinese University of Hong Kong, Hong Kong, China

**Keywords:** allergic rhinitis, Chinese herbal formula, nasal epithelium, gut microbiome, metabolome, network pharmacology

## Abstract

Allergic rhinitis (AR) is a series of allergic reactions to allergens in the nasal mucosa and is one of the most common allergic diseases that affect both children and adults. Shi-Bi-Lin (SBL) is the modified formula of Cang Er Zi San (CEZS), a traditional Chinese herbal formula used for treating AR. Our study aims to elucidate the anti-inflammatory effects and mechanisms of SBL in house dust mite-induced AR by regulating gut microflora metabolism. In vivo studies showed that nasal allergies and the infiltration of inflammatory cells in the nasal epithelium were significantly suppressed by SBL. Moreover, SBL restored the impaired nasal epithelial barrier function with an increased tight junction protein expression and reduced the endothelial nitric oxide synthase (eNOS). Interestingly, SBL significantly reconstituted the abundance and composition of gut microbiota in AR mice; it increased the relative abundance of potentially beneficial genera and decreased the relative abundance of harmful genera. SBL also restored immune-related metabolisms, which were significantly increased and correlated with suppressing inflammatory cytokines. Furthermore, a network analysis and molecular docking indicated IL-6 was a possible target drug candidate for the SBL treatment. SBL dramatically reduced the IL-6 level in the nasal lavage fluid (NALF), suppressing the IL-6 downstream Erk1/2 and AKT/PI3K signaling pathways. In conclusion, our study integrates 16S rRNA sequencing, microflora metabolism, and network pharmacology to explain the immune mechanism of SBL in alleviating HDM-induced allergic rhinitis.

## 1. Introduction

Allergic rhinitis (AR) is an allergic inflammatory disorder of the nasal mucosa characterized by rhinorrhea, sneezing, nasal congestion, and pruritus [1,2]. AR is mediated by the specific immunoglobulin (Ig) E and type 2 T helper (Th2) cell-dominant immune response. AR is usually triggered by environmental allergens, such as house dust mites (HDMs), cockroaches, animal dander, plant pollen, and mold [3]. Nasal epithelial cells are the crucial physical barrier in preventing allergens from entering the nasal tissue. The integrity of tight junctions (TJs) between nasal epithelial cells is essential for maintaining epithelial barrier function [4,5]. TJs are the most apically intercellular junctions through epithelial cells and include different transmembrane proteins (i.e., zonula occludens (ZO) family and occludin) [6,7]. In patients with HDM-induced AR, the mRNA expression level of ZO-1 and occludin protein expression in biopsy specimens was lower than in healthy control subjects [8]. As for the ex vivo and in vitro studies, it showed that ZO-1 and occludin expression were downregulated in the HDM-induced AR patients, causing an impaired barrier function compared with the healthy controls [8].

AR patients rely mainly on antihistamines, anti-leukotriene, glucocorticoids, and allergen-specific immunotherapy (desensitization). Antihistamines, anti-leukotriene, and glucocorticoids are common therapy strategies for allergic rhinitis, which can only temporarily relieve rhinitis symptoms and have associated side effects [9]. Allergen-specific immunotherapy (desensitization) induces immunotolerance to reach effective immune tolerance for a long time, but it would need more than 3 years to achieve the desired curative effect [10,11]. Therefore, it is necessary to develop an effective, safe, and long-term modality for the treatment of AR, and some Traditional Chinese Medicine (TCM) formulas have been demonstrated to have immunomodulatory effects for the treatment of AR with lower side effects [12]. TCM can be a promising candidate for AR therapy [13]. We and other groups have shown that Huanggui Tongqiao granules and Pentaherbs formula can significantly relieve the rhinitis symptoms, mitigate the infiltration of inflammatory cells in the nasal mucosa, and balance the Type 1 helper T cell (Th1)/Th2 ratio in the ovalbumin (OVA)-induced AR mouse model [12,14]. In the OVA-induced AR Hartley guinea pig model, the Yiqi Jiamin decoction can significantly decrease the infiltration of eosinophils and mast in the nasal mucosa [1]. Shi-Bi-Lin (SBL) constituted six Chinese medicines, *Xanthii Fructus*, *Angelicae Dahuricae Radix*, *Saposhnikoviae Radix*, *Magnoliae Flos*, *Gentianae Radix et Rhizoma*, and *Verbenae Herba*, which belong to the Jia Wei Cang Er Zi San (JW-CEZS) formula. JW-CEZS is the modified version of Cang Er Zi San (CEZS), which has been used for treating AR for more than 700 years [15]. SBL can significantly suppress the in vitro release of interleukin (IL)-4, IL-6, and TNF-α from the human mast HMC-1 cell line [15]. For the in vivo study, SBL significantly ameliorated the rhinitis symptoms, including sneezing and rubbing the nose and the production of IgG1, and suppressed the eosinophil infiltration and protein expression eNOS in the nasal tissue of the OVA-induced AR Hartley guinea pig model [13]. In a randomized, double-blind, placebo-controlled clinical trial, SBL was shown to be a safe and effective treatment for perennial AR with the suppression of rhinitis symptoms (such as nose blockage, sneezing, nose itching) [16]. However, the detailed immune-modulatory role and underlying mechanism of SBL in AR remain elusive.

Gut microbiota has been reported to be associated significantly with metabolic pathways and health outcomes [17,18]. The rural environment could modulate the gut microbiota to reduce allergies in children essentially [19]. The Shihu extract could modulate gut flora dysbiosis and increase the diversity and abundance of the intestinal flora in the OVA-induced AR mice model [20]. The Jia-Wei-Si-Miao-Yong-An decoction can improve cardiac function and reduce inflammation by altering the gut microbiota and their metabolism in the acute coronary syndrome rat model [21]. These studies suggested that the gut microbiota and its derived metabolites play an important role in disease development. TCM may modulate the gut microbiota and metabolites to relieve the disease symptoms [22]. Although TCM has gained more attention worldwide due to its long history and rich clinical experience, the complex compositions of TCM formulae have made it challenging to discover the potential and precise molecular mechanism [23]. 

The purpose of our study was to explore the anti-inflammatory effect and mechanism of SBL in AR by using 16S rRNA sequencing and metabolomics to identify the immunomodulating activities and potential molecular mechanism of SBL on the HDM-induced AR mice model, and it further predicted the potential mechanism of SBL in treating AR using network pharmacology, which was confirmed by the experimental approach.

## 2. Results

### 2.1. Identification of Marker Compounds of SBL by UPLC Analysis

**Figure 1:**
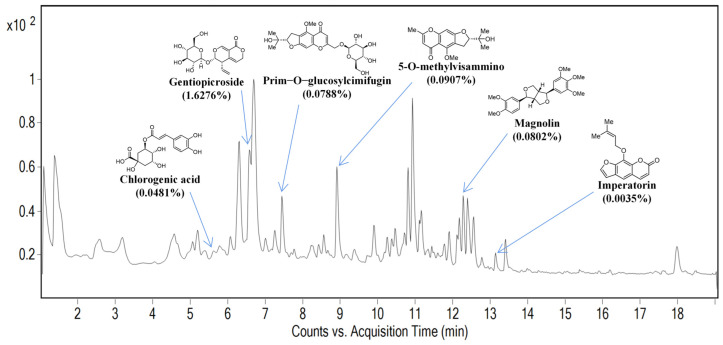
The UPLC chromatograms of SBL. The UPLC analysis was conducted using an Agilent 1290 UHPLC system coupled with a 6350 QTOF system (Santa Clara, CA, USA) and an Agilent ZORBAX Eclipse Plus RRHD column (2.1 mm × 150 mm, 1.8 μm). The chromatographic separation was maintained at 40 °C with gradient conditions at a 0.4 mL/min flow rate. Elution was performed with a mobile phase of A (0.1% formic acid in deionized and distilled water) and B (0.1% formic acid in acetonitrile) under a gradient program of 95% A at 0–2 min, 70–95% A at 2–9 min, 48–70% A at 9–10 min, 32–48% A at 10–12 min, 20–32% A at 12–13 min, and 20% A at 13–17 min, followed by 0–20% A at 17–19 min. Data were collected between 1 and 19 min.

The powder of the SBL decoction was dissolved in 1 mL methanol and diluted into 5 mg/mL for injection into Agilent 1290 UHPLC with a 6530 QTOF system. Figure 1 shows the LCMS profiles of SBL and the quantification of marker compounds of chlorogenic acid, gentiopicroside, prim-O-glucosylcimifugin, 5-O-methylvisamminol, magnolin, and imperatorinis.

### 2.2. SBL Treatment Alleviated Nasal Symptoms in HDM-Induced AR Mouse Model

**Figure 2:**
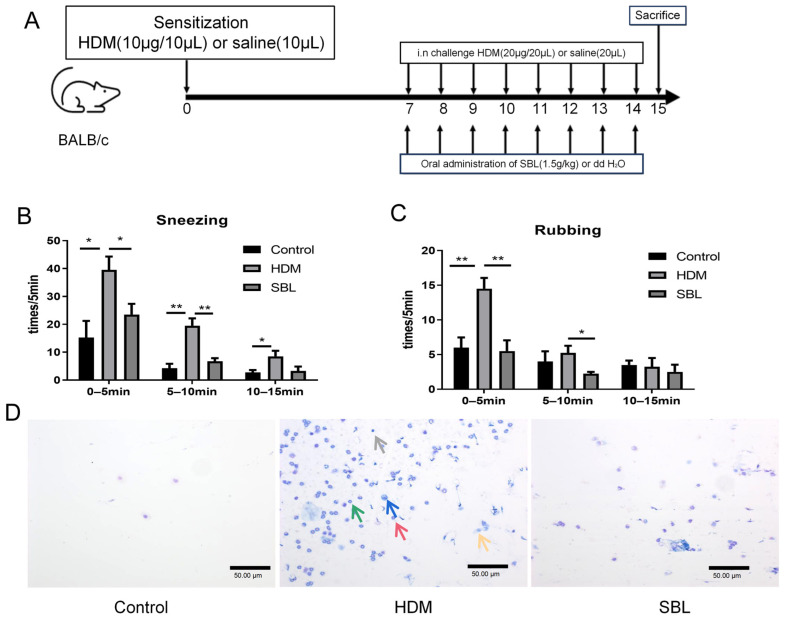

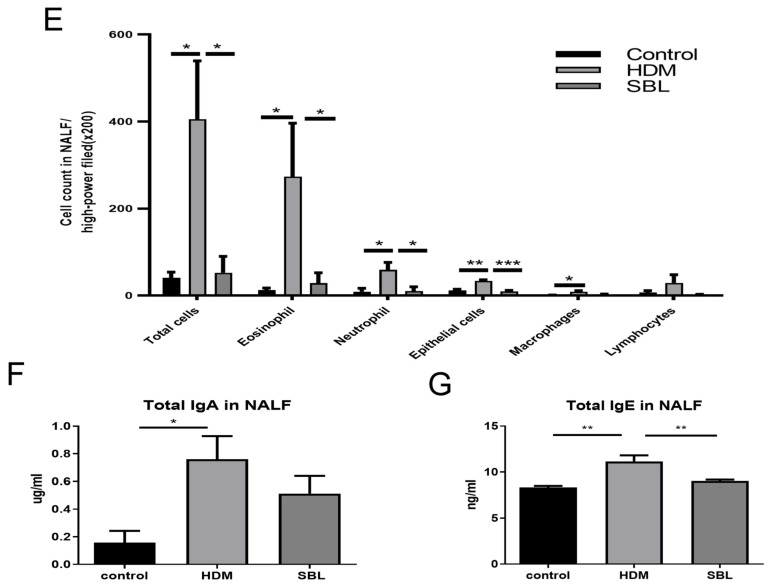
The effect of the oral treatment of SBL in an HDM-induced AR murine model (n = 3–4). (**A**) The schematic diagram of oral treatment with SBL in the HDM-induced AR mice; The frequency of sneezing (**B**) and nose rubbing (**C**) were recorded immediately in the first, second, and third 5 min intervals after the eighth HDM challenge on day 14. The infiltration of differential inflammatory cells and nasal epithelial cells in the NALF were stained with a Shandon Rapid-Chrome Kwik Diff Staining Kit (200×, with red arrows denoting the distribution of eosinophils, green arrows denoting the distribution of neutrophils, yellow arrows denoting the distribution of epithelial cells, blue arrows denoting the distribution of macrophage, gray arrows denoting the distribution of lymphocytes) (**D**); the number of total cells, eosinophil, neutrophils, nasal epithelial cells, macrophages, and lymphocytes were counted and presented in the bar charts (**E**). Total IgA (**F**) and total IgE (**G**) levels in the NALF were measured by ELISA. The results were presented with mean + SEM, * *p* < 0.05, ** *p* < 0.01, *** *p* < 0.001. Control: healthy control; HDM: HDM-induced AR mice; SBL: SBL oral treatment on HDM-induced AR mice.

To investigate the therapeutic effect of SBL in vivo, an AR mice model was established by intranasal sensitization and challenge by HDMs according to the SBL dose, which was converted from human to mice in a previous study [10,12] (Figure 2A). The sneezing and nose-rubbing frequencies after the eighth HDM challenge were recorded. The mice started to sneeze immediately upon the HDM challenge, and the peak frequency of sneezing and nose rubbing occurred in the first 5 min after the challenge. During the second and third 5 min periods, sneezing and nose rubbing frequencies decreased rapidly. The intragastric administration of SBL significantly reduced the frequency of sneezing and nose rubbing during the first and second 5 min periods compared with the HDM-induced AR control group (*p* < 0.05, Figure 2B,C). In the third 5 min, lower frequencies of sneezing and nose rubbing were shown in the HDM group, although no significant difference was demonstrated when compared with the SBL-treated group. The results showed that SBL substantially alleviated the sneezing and nose-rubbing symptoms in the HDM-induced AR mice model.

### 2.3. Oral Treatment of SBL Decreases the Infiltration of Different Inflammatory Cells, Nasal Epithelial Cells, and Immunoglobulin in the Nasal Lavage Fluid (NALF)

A total number of cells, eosinophils, neutrophils, epithelial cells, macrophages, and lymphocytes in the NALF were quantified by Kwik Diff Staining (Figure 2D,E). In the HDM-induced AR group, the total cell population, eosinophils, neutrophils, epithelial cells, and macrophages were increased compared with the healthy control group (All *p* < 0.05). After the oral treatment of SBL, the population of total cells, eosinophils, neutrophils, and epithelial cells decreased (All *p* < 0.05). At the same time, there was no significant difference in the cell number of macrophages and lymphocytes (*p* > 0.05). Total IgA (Figure 2F) and total Ig E (Figure 2G) levels in the NALF significantly increased in the HDM-induced AR group compared with that in the healthy control group. The HDM-specific IgE and IgG1 levels in the NALF also decreased with the SBL treatment (Figure S1A,B). Intragastrical administration with SBL markedly suppressed the total IgE levels in the NALF.

### 2.4. SBL Reduced Nasal Mucosa Histopathological Injuries and Nitric Oxide Protein in the HDM-Induced AR Mouse Model

**Figure 3:**
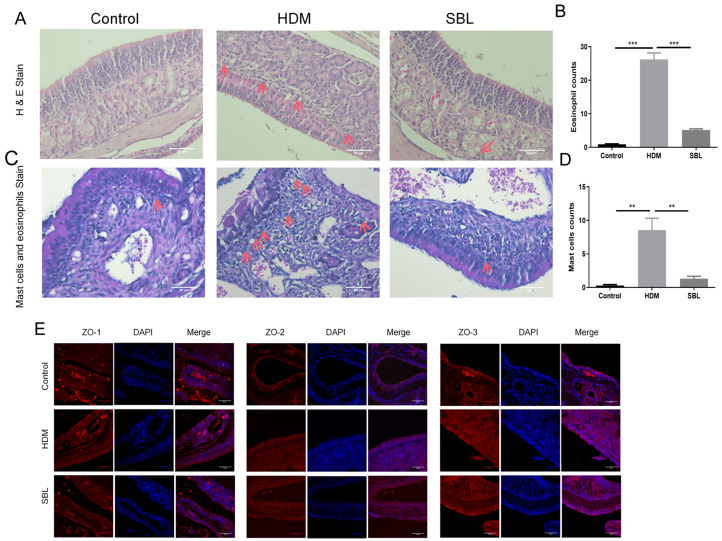

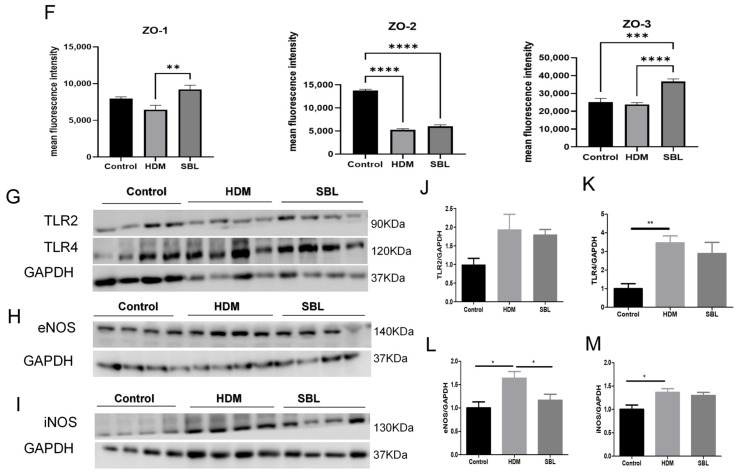
The attenuation of nasal mucosal tissue inflammation in HDM-induced AR mice upon SBL treatment (n = 4). (**A**) Representative hematoxylin and eosin staining (H and E, 200×, with red arrows denoting the distribution of eosinophils) of the sinonasal mucosa of the AR murine model with/without SBL treatment, with red arrows denoting the eosinophil infiltration in the nasal mucosa. (**B**) Counts of eosinophil infiltration at the nasal mucosal. (**C**) Staining of the sinonasal mucosa of the AR murine model with/without SBL treatment was performed using the Eosinophil–Mast Cell Stain Kit (200×, with red arrows denoting the distribution of mast cells). (**D**) Counts of mast cell infiltration at the nasal mucosal. (**E**) The comparison of ZO-1, ZO-2, and ZO-3 protein expression in nasal tissue among groups, with red representing ZO-1, ZO-2, or ZO-3 in different groups and blue representing DAPI. (**F**) Quantitative fold change in the mean fluorescence intensity of ZO-1, ZO-2 and ZO-3 expression. (**G**) Protein expression levels of TLR2 and TLR4 and (**H**,**I**) protein expression levels of eNOS and iNOS in the nasal mucosa of different groups were presented in a Western blot. Semi-quantitative analysis of (**J**) TLR2, (**K**) TLR4, (**L**) eNOS, and (**M**) iNOS levels based on grey-scale values of GAPDH. The results were presented with mean + SEM (n = 4). * *p* < 0.05, ** *p* < 0.01, *** *p* < 0.001, **** *p* < 0.0001. Control: healthy control; HDM: HDM-induced AR mice; SBL: SBL oral treatment on HDM-induced AR mice.

The gross morphology of nasal mucosa and infiltration of eosinophils and mast cells were examined using HE stains and a mast cell and eosinophil staining kit (Figure 3A,C). Eosinophils and mast cell infiltration in the HDM group were significantly higher than in the healthy control group (*p* < 0.05). After the oral treatment of SBL, the number of eosinophils and mast cells infiltrated considerably decreased (*p* < 0.05, Figure 3B,D). To investigate the tight junction protein expression level in the nasal mucosa, we determined ZO-1, ZO-2, and ZO-3 expression by immunofluorescent staining (Figure 3E,F). With the HDM challenge, the expressions of ZO-1, ZO-2, and ZO-3 in nasal mucosa were altered. However, SBL treatment increased the expression of ZO-1 and ZO-3 in the nasal tissue. Western blots were performed to clarify further the molecular action of SBL in the HDM-induced AR mice model and reveal the signaling pathway involved. The protein levels of TLR4 were increased in the AR mice compared to those in the healthy control mice, which indicated that HDMs might induce AR by activating TLR4 downstream signaling molecules (*p* < 0.05, Figure 3G,J,K). Finally, HDMs increased the nitric oxide protein levels of eNOS and iNOS, but SBL significantly restored the protein level of eNOS (*p* < 0.05, Figure 3H,I,L,M).

### 2.5. Effect of SBL on Gut Microbiota Profiles in HDM-Induced AR Mice 

**Figure 4:**
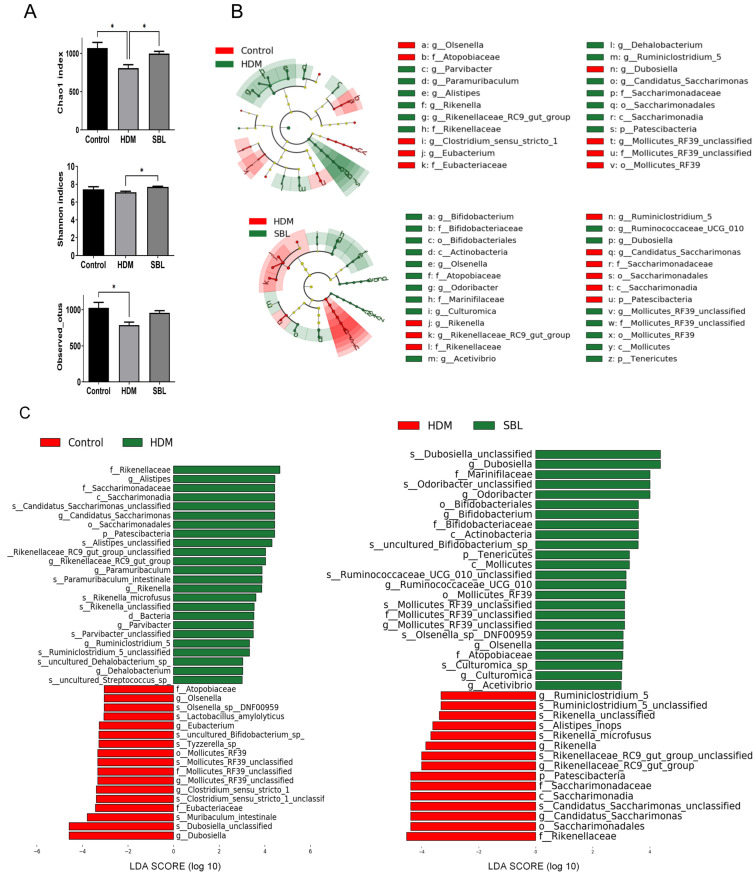
The effect of SBL treatment on the diversity and composition of gut microbiota (n = 6). (**A**) Alpha diversity includes the Chao1 index, Shannon indices, and Observed OTUS. (**B**) The cladogram showed significant changes in gut microbiota between the healthy control group and the HDM group as well as between the HDM group and the SBL-treated group. (**C**) LEfSe results comparing the fecal microbiota between the healthy control and HDM groups, with a linear discriminant analysis (LDA) score of greater than +3.0, and between the HDM and SBL-treated groups, with an LDA score of greater than +3.0, as represented by the length of the bar. * *p* < 0.05. Control: healthy control; HDM: HDM-induced AR mice; SBL: SBL oral treatment on HDM-induced AR mice.

To further explore the potential role of SBL on intestinal homeostasis, we performed 16S rRNA sequencing on AR mice fecal samples to investigate the gut microbiota changes upon SBL treatment. The alpha diversity, including the Chao 1 index, Shannon index, and Observed OTUs, depicted the diversity and abundance of gut microbiota (Figure 4A). Compared with the healthy control group, the HDM group showed a dramatically reduced Chao 1 index, which is an operational taxonomic unit parameter for microbiota species richness [23]; SBL treatment could significantly increase the Chao 1 index compared with the HDM group (*p* < 0.05). The SBL-treated group displayed a significant restoring effect for the quantitative Shannon index compared with the HDM group (*p* < 0.05). These findings showed that SBL treatment could increase gut microbial diversity and uniformity. However, in the Observed OTUS, SBL could alleviate the suppression effect by the HDMs without significant differences found among the groups (*p* > 0.05, Figure 4A).

**Figure 5:**
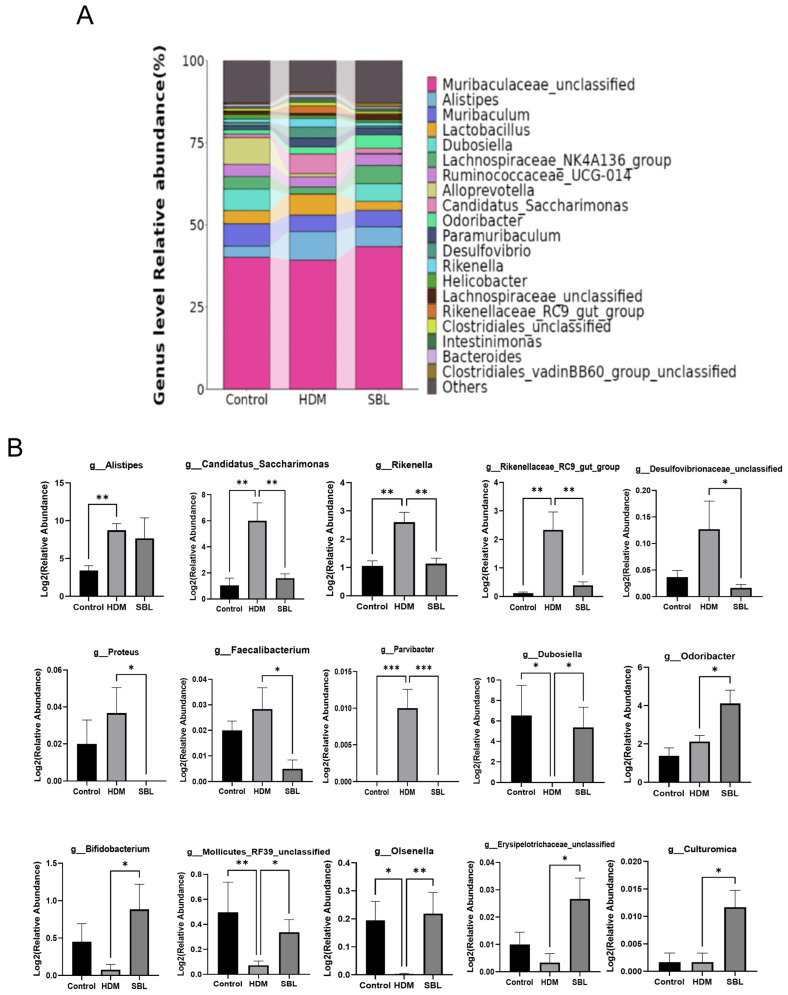
The effect of SBL on the gut microbiota disturbance in HDM-induced AR mice (n = 6). (**A**) The taxonomy classification of gut microbiota at the genus level among different groups displayed with the top 20 enriched class categories. (**B**) The abundance of differential bacteria in the three groups at the genus level was shown. The results were presented with mean + SEM. * *p* < 0.05, ** *p* < 0.01, *** *p* < 0.001. Control: healthy control; HDM: HDM-induced AR mice; SBL: SBL oral treatment on HDM-induced AR mice.

To clarify the effect of SBL and HDMs on the composition and structure of gut microbiota, we profiled the relative abundance of the top 20 gut microbiota at the family level (Figure S2A), phylum level (Figure S2B), and genus level (Figure 5A) to further investigate the species changes. At the family level, the Muribaculaceae, Rikenellacea, Ruminococcsceae, Lachnospiraceae, and Lactobacillaceae were the most abundant species (Figure S2A). There was no significant difference at the family level among the control, HDM, and SBL groups. At the phylum level, Bacterioidetes, Firmicutes, and Patescibacteria account for more than 90% of the identified microbiota in all treatment groups, and the composition of gut microbiota in the HDM group was varied while compared with a healthy control group and SBL treatment group (Figure S2B). Compared with the healthy control group, the HDM group showed a lower proportion of phyla Cyanobacteria and a higher proportion of *Patescibacteria* (Figure S2C). At the genus level, after the oral treatment of SBL in the HDM-induced AR mice, the proportion of gut microbiota was similar to the healthy control group and the relative abundances of potentially beneficial genera (e.g., *Dubosiella* and *Olsenella*), while the proportions of harmful genera (e.g., *Rikenella* and *Rikenellaceae_RC9_gut_group*) were significantly reduced when compared with the HDM group. The bar chart performed a differential analysis of gut microbiota at the phylum level (Figure 5B). 

To further ascertain the key bacterial taxa in gut microbiota between the groups, the linear discriminant analysis (LDA) effect size (LEfSe) analysis was performed (Figure 4B,C). As shown in Figure 4B, regarding the relative abundance of in the cladogram, from the outside to inside, the layer represents the classification level from species to kingdom, and the size of the small circle denotes the corresponding abundance of the bacteria. The results suggested that *Patescibacteria* at the phylum level and *Candidatus_Saccharimonas, Mollocutes_RF39_unclassified* and *Rikenellaceaw_RC9_gut_group* et al. at the genus level, were the essential taxa in the HDM-induced AR development in the mice model. SBL treatment could restore the *Patescibacteria* at the phylum level and *Bifidobacterium, Candidatus_Saccharimonas*, and *Rikenellaceaw_RC9_gut_group* et al. at the genus level in the AR mice (Figure 5A,B).

### 2.6. Oral Treatment of SBL Altered the Gut Metabolites in HDM-Induced AR Mice 

**Figure 6:**
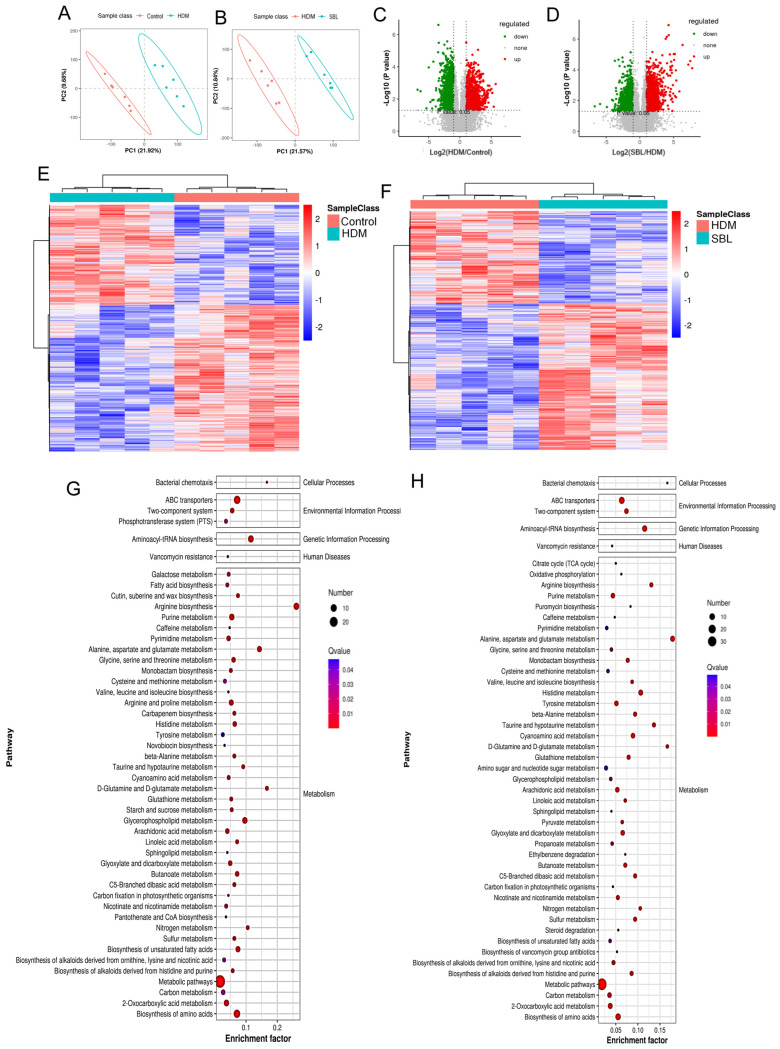

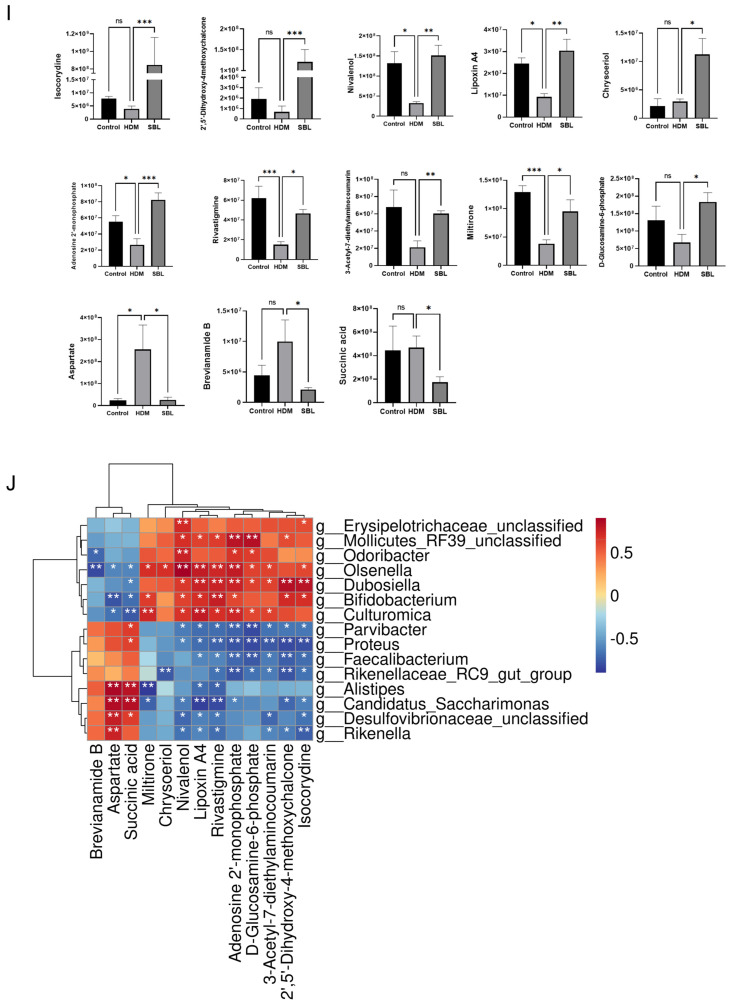
Alteration in gut microbiota-related metabolites in HDM-induced AR mice upon SBL treatment (n = 6) and correlations of microorganisms at the genus level and metabolites in fecal samples. The principal component analysis (PCA) plot of the gut metabolomic analysis in healthy control mice and HDM-induced AR mice (**A**) as well as in HDM-induced AR mice and HDM-induced AR mice with SBL treatment (**B**). A Volcano plot of differential metabolites between the healthy control mice and HDM-induced AR mice (**C**) and between the HDM-induced AR mice and SBL-treated AR mice (**D**). In the Volcano plots, red dots represent the significantly upregulated metabolites, blue dots represent the significantly downregulated metabolites, and grey dots represent the metabolites without significant changes. The metabolic ion heatmap with red and blue blocks indicates the increase and decrease in relative abundance between the healthy control mice and HDM-induced AR mice (**E**) and between the HDM-induced AR mice and SBL-treated HDM-induced AR mice (**F**). KEGG-enriched pathways of differential metabolites between the healthy control mice and the HDM-induced AR mice (**G**) and between the HDM-induced AR mice and the HDM-induced AR mice (**H**). An abundance comparison of key metabolites among the three groups (**I**). In the Pearson correlation heatmap between the microbiota genera and the altered metabolites between the HDM and SBL groups, red denotes a positive correlation, and blue denotes a negative correlation (**J**). Statistical significance (*p* < 0.05) is visually depicted from red to purple, where red represents the greatest difference and purple the least. ns: non-significant, * *p* < 0.05, ** *p* < 0.01, *** *p* < 0.001. Control: healthy control; HDM: HDM-induced AR mice; SBL: SBL oral treatment on HDM-induced AR mice.

The gut microbiota strongly influences the fecal metabolites. A partial least squares-discriminant analysis (PLS-DA) was used to find out the difference in metabolites between the healthy control group and the HDM group (Figure 6A) and between the HDM group and the SBL-treated group (Figure 6B). The wide separation and spatial distribution suggested that fecal metabolites were altered after HDM or SBL treatment. Between the HDM group and the healthy control group, a total of 17,100 positive ion-mode metabolite changes were identified, including 994 significantly over-expressed metabolites and 690 considerably downregulated metabolites, and a total of 15,229 negative ion-mode metabolite changes were identified, including 876 significantly over-expressed metabolites and 834 considerably downregulated metabolites (Figure 6C,E, Supplementary File S2). Between the SBL-treated group and the HDM group, a total of 17,100 positive ion-mode metabolite changes were identified, including 913 significantly over-expressed metabolites and 625 considerably downregulated metabolites, and a total of 15,229 negative ion-mode metabolite changes were identified, including 836 significantly over-expressed metabolites and 829 considerably downregulated metabolites (Figure 6D,F, Supplementary File S2).

To further investigate the metabolic pathways affected explicitly by the HDM challenge and SBL treatment, a Kyoto Encyclopedia of Genes and Genomes (KEGG) pathway analysis was performed to reveal the differentially expressed metabolite pathways involved between the healthy control group and HDM-induced AR group (Figure 6G) and between the HDM-induced AR group and SBL-treated HDM-induced AR group (Figure 6H). A total of 48 KEGG pathways were identified in each group, which include cellular processing, environmental information processing, genetic information processing, and metabolism, such as the citrate cycle, metabolism, and biosynthesis of amino acids, lipids, and carbohydrates. Most differential KEGG pathways were associated with the synthesis and metabolism of amino acids and aminoacyl-tRNA biosynthesis.

The representative metabolites identified from significantly different metabolites and the KEGG pathway was shown in (Figure 6I), including isocorydine, 2′,5′-Dihydroxy-4-methoxychalcone, 3,3-Dimethylglutaric acid, nivalenol (NIV), chrysoeriol, lipoxin A4, adenosine 2′-monophosphate, rivastigmine, 3-Acetyl-7-diethylaminocoumarin, D-Glucosamine-6-phosphate, miltirone, succinic acid, and aspartate. Importantly, NIV showed the inhibition effect on the antigen-specific Th2 response upon the oral administration of OVA [24]. Isocorydine and rivastigmine have been reported to suppress inflammatory cytokine IL-6, IL-1β, and TNF-α in lipopolysaccharide (LPS) treatment macrophages [25,26,27]. Chrysoeriol has been reported to have anti-inflammatory and anti-oxidative effects in several in vitro studies, and lipoxin A4 has anti-inflammatory and immunoregulatory effects in LPS-induced sepsis [28,29,30]. Inversely, the aspartate could promote inflammatory cytokine production in IL-1β by boosting the hypoxia-inducible factor (HIF)-1α in the LPS and IFN-γ co-stimulated M1 macrophage [31]. The succinic acid was the innate immune signaling to increase the IL-1β by HIF-α [32].

To better understand the relationship between gut microbiota and fecal metabolites, we performed the correlation analysis between bacterial genera and representative metabolites between HDM-treated and SBL groups (Figure 6J). From the correlation analysis, we found that a specific gut microbiota was significantly correlated with representative metabolites. The increase in anti-inflammatory-related metabolites, such as isocorydine, 2′,5′-Dihydroxy-4-methoxychalcone, 3,3-Dimethylglutaric acid, NIV, chrysoeriol, lipoxin A4, adenosine 2′-monophosphate, rivastigmine, 3-Acetyl-7-diethylaminocoumarin, D-Glucosamine-6-phosphate, miltirone, and 11−Dehydrocorticosterone, shows a significant positive correlation with the beneficial bacterial genera, including Dubosiella, Olsenella, and Mollicutes_RF39_unclassified, and a negative correlation to deleterious bacterial genera, including Alistipes, Desulfovibrionaceae_unclassified, and *Rikenella.* In contrast, disease-related metabolites, such as aspartate and succinic acid, had a significant positive correlation with deleterious bacterial genera and a negative correlation with beneficial bacterial genera.

### 2.7. Network Pharmacology Analysis

**Figure 7:**
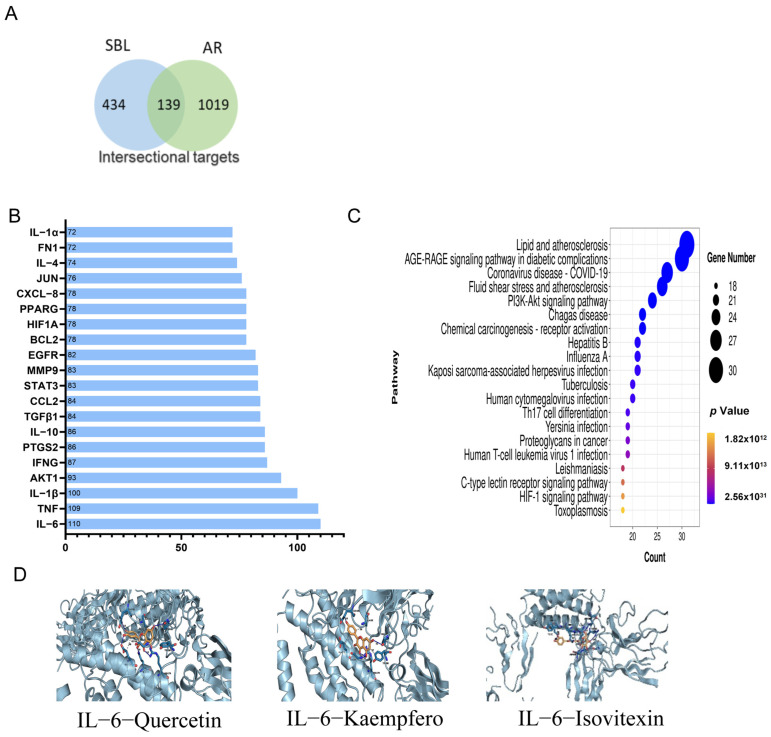
SBL network pharmacology and the functional analysis. (**A**) The Venn graph of the intersection of SBL and AR. (**B**) The bar graph of the top 20 critical targets in the node degree. (**C**) The bubble map of the top 20 signaling pathways related to the effect of SBL in the treatment of AR based on the KEGG enrichment analysis. The X-axis and Y-axis show the enrichment score and full names of the processes, respectively, and the color and size of each bubble represent the *p*-value and gene count, respectively. (**D**) The molecular docking of active ingredients IL-6.

Seventy-six potential active ingredients of SBL were obtained from the TCMSP database; all the ingredients fulfilled the ADME parameters and Lipinski’s rule. There were 573 related targets of different ingredients obtained from TCMSP or predicted from the Swiss Target Prediction platform. After the screening of GeneCards, the OMIM database, the Therapeutic Target Database, and the DisGeNET database using the keywords “Allergic Rhinitis” and “Rhinitis, Allergic”, there were 1158 targets identified. After the intersection of conditions, 139 potential targets intersected the AR and SBL (Figure 7A).

The Cytoscape (version 3.10.2) was used to construct the ingredient-target network (Figure S3A). Here, 68 active ingredients from SBL and 139 targets in AR intersected in the compound–target interaction network. The 139 targets were imported to the STRING database to construct the PPI network and visualized by Cytoscape (version 3.10.2) (Figure S3B). The edges represented the interactions between the target protein. The top 20 targets are shown in Figure 7B. The IL-6 is the top target based on the node degree.

In the GO function analysis, a total of 456 items in three categories were identified: 194 biological processes (BP), 294 molecular functions (MF), and 68 cellular components (CC). The top 10 enriched BP terms, MF terms, and CC terms are presented in the column chart. In the KEGG enrichment analysis, the 139 potential targets were highly enriched for 258 pathways by filtering them with *p* < 0.05 as the significance level and count >2; 162 pathways were selected. The top 20 highly enriched pathways were indicated in the column chart (Figure 7C). IL-6 participated in 15 signaling pathways. Therefore, IL-6 was essential for intersection targets.

Based on the degree of the PPI network, GO, and the KEGG analysis, IL-6 was selected for molecular docking. There are three active gradients, quercetin (MOL000098), isovitexin (MOL0002322), and kaempferol (MOL000422), that have stronger binding activity with IL-6, which have binding energies of −9 kcal/mol, −9.1 kcal/mol, and −8.8 kcal/mol, respectively (Figure 7D). The binding affinity between IL-6-quercetin, IL-6-isovitexin, and IL-6-kaempferol was lower than −8.0 kcal/mol, indicating a stable binding affinity between the target’s protein and the potential active component.

### 2.8. Alleviation of IL-6 Level in NALF and Suppression of the Erk and PI3K/AKT Signaling in Nasal Mucosa Upon SBL Treatment

**Figure 8:**
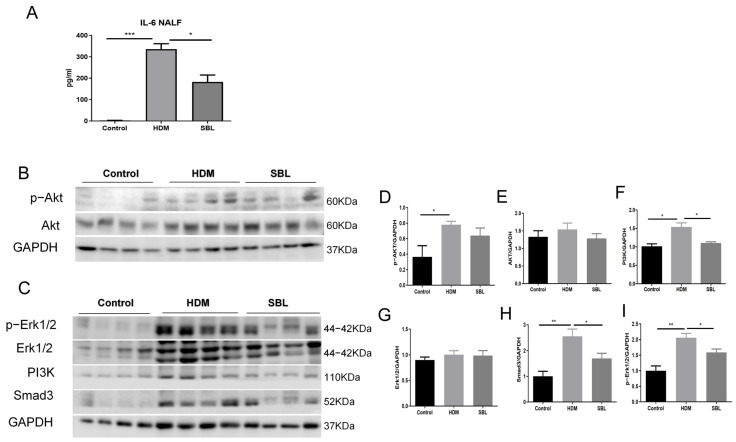
The alleviation of the IL-6 level in the NALF and suppression of the Erk and PI3K/AKT signaling in nasal mucosa upon SBL treatment. (**A**)The levels of inflammatory cytokines IL-6 in the NALF were determined using CBA with flow cytometry (BD FACSVia flow cytometer); (**B**,**C**) protein expression levels of the expression of signaling molecules (p-AKT, AKT, p-Erk1/2, Erk1/2, PI3K, and Smad3) in the nasal mucosa of different groups were presented in a Western blot. The semi-quantitative analysis of (**D**) p-AKT, (**E**) AKT, (**F**) p-Erk1/2, (**G**) Erk1/2, (**H**) Smad3, and (**I**) PI3K levels based on grey-scale values of GAPDH. Results were presented with mean + SEM. * *p* < 0.05, ** *p* < 0.01, *** *p* < 0.001. Control: healthy control; HDM: HDM-induced AR mice; SBL: SBL oral treatment on HDM-induced AR mice.

To further confirm the mechanism of SBL in HDM-induced AR mice, the IL-6 level in the NALF was quantified. Upon the HDM treatment, the IL-6 (Figure 8A) level in the NALF was significantly increased compared with that in the healthy control group, while oral administration with SBL markedly suppressed IL-6 levels in the NALF. Furthermore, HDMs significantly phosphorylated both AKT and Erk1/2, and SBL suppressed the phosphorylation level of Erk1/2 and the protein expression level of PI3K and Smad3 in nasal epithelium tissue (all *p* < 0.05, Figure 8B–I).

## 3. Discussion 

An HDM is an indoor allergen, which is the most common allergen to trigger perennial AR and plays a vital role in the development of a series of allergic diseases, including AR, AD, and asthma [33]. Different from the previous study, which preliminarily clarified the mechanism of SBL for AR therapy with the OVA-induce Hartley guinea pigs model, in our study, we further evaluate the anti-allergic and anti-inflammatory effect of the SBL formula in the most common allergen-HDM-induced AR (Figure 2A) [13]. 

Before establishing the AR mouse model with HDMs, we identified and quantified the marker compounds of SBL with a UPLC analysis for quality control, including chlorogenic acid, gentiopicroside, prim-O-glucosylcimifugin, 5-O-methylvisamminol, magnolin, and imperatorin [16]. We found that SBL could alleviate the rhinitis symptoms of nasal sneezing and nose rubbing, which are the primary diagnosis of AR [34]. SBL could significantly inhibit the in situ release of total IgA and IgE in the NALF, reduce the infiltration of inflammatory cells in the nasal tissue, and maintain the integrity of the nasal epithelial barrier by upregulating the protein expression of tight junction proteins and downregulating the eNOS. In AR patients, the IgA levels would increase in the nasal mucosa similarly to the IgE and correlate with the nasal symptoms [35]. SBL would slightly lessen the IgA level in the NALF, although no significant difference was shown. Nevertheless, SBL could reduce inflammatory cells, such as eosinophils, neutrophils, and mast cells, and prevent damage to the nasal epithelial barrier in the nasal mucosa. The accumulation of eosinophils, associated with the release of Th2 cytokines IL-4, IL-5, and IL-13, is a characteristic of inflammatory diseases [12,34]. As for the mast cells, it not only induces a histamine release from the nasal mucosa but also releases chemotactic factors to promote the infiltration of immune cells to the local inflammatory site to induce an immune response [36]. In our study, we show that the HDM could increase the protein expression level of bacterial TLR4 to activate further downstream signaling pathways, which subsequently causes the increase in nitric oxide iNOS and eNOS [30,37,38,39,40,41]. Nitric oxide can cause nasal congestion by inducing the vessel dilatation and hyper-function of submucosal glands. Therefore, it plays an essential role in AR disease [13]. Although SBL does not significantly decrease the protein expression level of TLR4, SBL can potentially reduce the eNOS level in the nasal mucosa. 

A growing number of studies indicate that microbiota dysbiosis is recognized as an essential factor in the development of allergic diseases [42]. The 16S rRNA sequencing of gut microbiota revealed that SBL could restore the microbiota dysbiosis in the HDM-induced AR mice by increasing the abundance and composition of intestinal flora, resulting in the restoration of the microbe’s diversity, as observed in the healthy control group at the genus level and phylum level. HDMs could change the proportion of *Aistipes*, *Candidatus_Saccharimonas*, *Rikenella*, *Desultfovibrionace_unclassified*, *Proteus*, *Mollocutes_RF39_unclassified*, and *Rikenellaceaw_RC9_gut_group* et al. at the genus level, which can trigger AR development in the mice model. 

Metabolomics has been increasingly applied in disease diagnosis, the investigation of disease mechanisms, the identification of multiple drug targets, and the evaluation of therapeutic outcomes [43,44]. The SBL formula comprises six herbal medicines that constitute complex macromolecules: carbohydrates, protein, protein-bound polysaccharides, and glucocorticoid (GC) [13]. In our study, oral treatment with SBL could regulate metabolism in 48 KEGG pathways, including cellular processing, environmental information processing, and metabolism. SBL increased the beneficial metabolites (e.g., isocorydine, NIV, chrysoeriol, lipoxin) and reduced the harmful metabolites (e.g., aspartate and succinic acid). Furthermore, there was a strong relationship between metabolite alterations and gut microbiota. The genera *Candidatus_Saccharimonas* and *Faecalibacterium* have been reported to increase in age-related macular degeneration (AMD) mice, which indicates that they would have an inflammatory effect and result in the immune response in the AMD [45]. In this study, *Candidatus_Saccharimonas* and *Faecalibacterium* increased in the HDM-induced AR mice; after the SBL treatment, their levels significantly decreased. Both had strong negative correlations with the anti-inflammatory-related metabolites (e.g., isocorydine, and rivastigmine) and significant correlation with the aspartate and succinic acid. Isocorydine and rivastigmine could suppress inflammatory cytokine IL-6, IL-1β, and TNF-α in lipopolysaccharide (LPS) treatment macrophages [25,26,27]. In contrast, all *Olsenella*, *Dubosiella*, and *Bifidobacterium* have been identified as beneficial genera for improving anti-inflammatory function [46,47,48]. They exhibited significantly positive correction with the anti-inflammatory metabolites, including chrysoeriol, nivalenol, rivastigmine, lipoxin A4, and adenosine 2′-monophosphate, and had a negative correlation with pro-inflammatory metabolites aspartate and succinic acid. 

By 16s RNA sequencing and a fecal metabolomic analysis, we have confirmed that SBL could modulate the composition and metabolomics of gut microbiota to play an anti-inflammatory role in allergic rhinitis. To further predict the potential mechanism and related pharmacological targets of SBL for AR treatment, network pharmacology and molecular docking were performed. A network pharmacology analysis is useful for integrating massive data to predict the therapeutic target of TCM ingredients and interested disease [49]. Notably, IL-6 participated in 15 signaling pathways from the top 20 KEGG pathways. From the molecule docking, IL-6 showed high binding activity with quercetin (MOL000098), isovitexin (MOL0002322), and kaempferol (MOL000422). Therefore, via a KEGG analysis and molecular docking validation, we inferred that IL-6 would be considered therapy targets of SBL for AR treatment. 

In our study, the IL-6 level in the NALF was significantly increased in the HDM-induced AR group compared with that in the healthy control group. At the same time, SBL markedly suppressed IL-6 levels in the NALF (Figure 8A). AKT and Erk are the downstream effectors of IL-6, and IL-6 production would be modulated by the activation of the Erk and AKT signaling pathway [50,51]. HDMs significantly phosphorylated both AKT and Erk1/2, and SBL suppressed the phosphorylation level of Erk1/2 and protein expression level of PI3K and Smad3 in nasal epithelium tissue. The pro-inflammatory cytokine IL-6 can promote the differentiation and activation of T cells and the subsequent release of Th2 cytokines in allergic diseases such as asthma, AD, and AR [46,47]. Allergen-induced IL-6 trans-signaling activity could increase inflammation and lung dysfunction by promoting the production of chemokines [52]. The HDM can significantly increase the secretion of IL-6 levels in the nasal cavity, and oral treatment with SBL could remarkably reduce IL-6 in the NALF. 

From the gut microbiota and metabolism studies, we found that SBL could increase the anti-inflammatory-related microbiota and metabolites and specially regulate the IL-6-related microbiota and metabolites. In an in vivo study, the IL-6 level in the NALF and the phosphorylation level of AKT and Erk1/2 in nasal tissue were significantly increased with the HDM. After SBL treatment, the IL-6 level in the NALF and the phosphorylation level of Erk1/2 were suppressed considerably. Although the phosphorylation level and total level of AKT were found with minor changes, the protein level of PI3K, an important signaling molecule in the AKT/PI3K pathway, significantly decreased. Previous studies also indicated that the inhibition of Erk and AKT/PI3K could upregulate the expression of ZO-1 in both mRNA and protein levels [44,45]. We can infer from this that the binding of IL-6 and active gradients from SBL would not affect the amount of AKT but affect related signaling molecules in the AKT/PI3K and Erk1/2 pathway. 

## 4. Materials and Methods

### 4.1. Preparations of Shi-Bi-Li (SBL)

As described in previous studies [10,12], SBL (150 g) comprised six raw herbal ingredients, including Xanthii Fructus 18.75 g, Angelicae Dahuricae Radix 50 g, Saposhnikoviae Radix 18.75 g, Magnoliae Flos 37.5 g, Gentianae Radix et Rhizoma 12.5 g, and Verbena Herba 12.5 g. The natural medicines were purchased from Zi Sun (Hong Kong, China) Limited, Kwai Chung, Hong Kong, and extracted by refluxing in boiling water as previously described [10]. Briefly, the SBL was prepared by mixing six herbs and refluxing them in 100 °C boiling water for 3 h; the mixture of the water crude extracts was filtered and condensed with a rotary evaporator before being lyophilized into powder. The quality of SBL was quantified by UPLC analyses (Figure 1). Each mouse was administrated with a dose of 1.5 g/kg (crude drug) according to the dose that has been converted from humans to mice in the previous study [10,12].

### 4.2. Animal Experiment

Pathogen-free BALB/c mice (aged 6–8 weeks, 15–20 g body weight) were obtained from the Laboratory Animal Services Centre, the Chinese University of Hong Kong (Hong Kong, China). All mice in this study were maintained and handled according to the CUHK Animal Experimentation Ethics Committee Guide for the Care and Use of Laboratory Animals. After a week’s accommodation, BALB/c mice were sensitized with 10 μg/10 μL HDMs (Greer Laboratories, Lenoir, NC, USA) or 10 μL saline by nasal drip on day 0. From day 7 to 14, SBL (0.75 g/kg, 1 g/kg, and 1.5 g/kg in 200 μL H_2_O) or 200 μL H_2_O were given to the mice by oral gavage. From day 7 to day 14, mice were intranasally challenged with 20 μg/20 μL HDMs or 20 μL saline one hour after oral SBL treatment. Stool samples were collected under pathogen-free conditions on day 15 and stored at −70 °C immediately. Mice were then euthanized on day 15 (Figure 2A).

### 4.3. Evaluation of Nasal Allergic Symptoms

On day 14, two observers, who were blinded, recorded the frequencies of sneezing and nose rubbing of each mouse during the first 15 min period immediately after the last HDM challenge. 

### 4.4. Collection of Nasal Lavage Fluid (NALF) and Quantification of Immunoglobulin and Cytokines 

The mice were sacrificed by high-dose carbon dioxide (CO_2_) on day 15. The trachea was exposed and ligated at the upper level. Then, cold PBS (1 mL) was gently instilled into the nasopharynx by a 21-gauge catheter to obtain NALF. NALF was centrifuged at 3000 rpm for 5 min at 4 °C to collect supernatant, which was stored at −70 °C to measure the cytokine level. The concentrations of murine total IgE and immunoglobulin A (IgA) in NALF were measured using an Enzyme-Linked Immunosorbent Assay (ELISA) kit (eBioscience, Hartfield, UK). Concentrations of murine IL-6 in NALF were measured using a multiplex LEGENDplex™ cytometric bead-based assay (CBA) kit (BioLegend Inc. San Diego, CA, USA) with BD FACSVia flow cytometer (BD Biosciences, San Jose, CA, USA). 

### 4.5. Determination of Nasal Epithelial Cells and Immune Cells in NALF

The cells in NALF were resuspended with 50 μL PBS and centrifuged onto glass slides by the cytospin (Centrifuge 5403, Eppendorf, Hamburg, Germany) and stained with Kwik Diff Staining kit (Thermo Fisher Scientific, Waltham, MA, USA). The numbers of total cells, eosinophils, neutrophils, lymphocytes, macrophages, and nasal epithelial cells were counted in five random 200× power fields per specimen using Leica DM6000B microscope (Leica Microsystems GmbH, Wetzlar, Germany) and the Leica Application Suite software (Leica Microsystems, Wetzlar, GmbH, Version: 3.2.0).

### 4.6. Histological Examination and Immunofluorescence (IF) Study

The nose of HDM-induced mice was fixed in 4% formalin for 24 h and decalcified with 10% ethylenediaminetetraacetic acid (EDTA) for 21 days before being dehydrated and embedded in paraffin for histopathological evaluation. Paraffin sections (5 μm) of sinonasal mucosa tissues were stained with hematoxylin and eosin (H and E) (Beyotime Institute of Biotechnology, Haimen, China) for the identification of infiltration eosinophils. Mast cells were stained with an Eosinophil–Mast Cell Stain Kit (Abcam, Cambridge, UK). Eosinophils and mast cells were counted manually at three different locations of each image.

For the immunofluorescence assay, paraffin sections (5 μm thickness) of sinonasal mucosa tissue were deparaffinized in xylene, rehydrated, and immunostained with ZO-1 primary antibody (Thermo Fisher Scientific, 1:100), ZO-2 primary antibody (Cell signaling technology, Danvers, MA, USA, 1:200), and ZO-3 primary antibody (Thermo Fisher Scientific, 1:200) overnight. Cy3-conjunction goat anti-rabbit IgG antibody (ABclonal, Woburn, MA, USA, 1:800) was used as a secondary antibody for 30 min staining.

### 4.7. Western Blot

Mice nasal mucosal tissues were homogenized and lysed in T-PER Tissue Protein Extraction Reagent (Thermo Fisher Scientific) containing protease inhibitors and phosphatase inhibitors (Sigma-Aldrich, St. Louis, MI, USA) for 30 min on ice. Proteins from tissue extraction were separated by SDS–PAGE gel and transferred to PVDF membranes (Millipore, Burlington, MA, USA). The PVDF membranes were blocked in 5% Bovine Serum Albumin (BSA) for 2 h at room temperature. They were then incubated with primary antibodies against toll-like receptor (TLR) 2 (Thermo Fisher Scientific, 1:1000), TLR4 (Abcam, Boston, MA, USA, 1:1000), GAPDH, p-Erk1/2, Erk1/2, p-AKT, AKT, PI3K, Smad3, eNOS, and inducible nitric oxide (iNOS) (Cell signaling technology, 1:1000) overnight at 4 °C. Secondary antibody (HRP-conjugated anti-rabbit IgG and HRP-conjugated anti-mouse IgG, Cell Signaling Technology) was used at a ratio of 1:5000 for 2 h at room temperature. The results were visualized using the Amersham enhanced chemiluminescence (ECL) Western blotting detection reagent kit (GE Healthcare, Chicago, IL, USA) and chemiluminescence imaging system (Syngene, Bangalore, India). Results were analyzed by Image J software (v1.51), and the relative expression level of the target protein was evaluated after normalizing with the housekeeping gene GAPDH.

### 4.8. 16S rRNA Sequence

According to the manufacturer’s instructions, total genome DNA from stool samples was extracted using the E.Z.N.A. ^®^Stool DNA Kit (D4015, Omega, Biel/Bienne, Switzerland). Extracted DNA (25 ng) was amplified using specific (16S V3–V4) primer (341F 5′-CCTACGGGNGGCWGCAG-3′, 805R 5′-GACTACHVGGGTATCTAATCC-3′). After that, the products were purified by AMPure XT beads (Beckman Coulter Genomics, Danvers, MA, USA) and quantified by Qubit (Invitrogen, Waltham, MA, USA). The amplicon pools were prepared for sequencing, and the size and quantity of the amplicon library were assessed on Agilent 2100 Bioanalyzer (Agilent Technologies Inc., Santa Clara, CA, USA) and the Library Quantification Kit for Illumina (Kapa Biosciences, Woburn, MA, USA), respectively. The libraries were sequenced on the NovaSeq PE250 platform.

Samples were sequenced on an Illumina NovaSeq platform according to the manufacturer’s recommendations, provided by LC-Bio Technology Co., Ltd. (Hangzhou, Zhejiang Province, China), as previously described [23]. In brief, paired-end reads were assigned to samples based on their unique barcode and were truncated by cutting off the barcode and primer sequence, and the reads were then incorporated using FLASH (28). According to fqtrim (V 0.94), clean tags were acquired by quality filtering on the raw reads. Chimeric sequences were filtered using Vsearch software (v2.3.4). The feature table and feature sequence were obtained after being dereplicated by DADA2. The species diversity of each sample was revealed by alpha diversity, while the differences in the complexity of species were evaluated by beta diversity. Alpha and beta diversity were calculated by randomly normalizing them to the same sequences with QIIME2. Referring to SILVA (release 132) classifier, feature abundance was normalized using the relative abundance of each sample. The blast was used for sequence alignment, and the feature sequences were annotated with the SILVA database for each representative sequence.

### 4.9. Metabolism Studies

The stool samples were collected on day15, extracted by 50% methanol buffer, and analyzed using ultra-high performance liquid chromatography–mass spectrometry (UPLC-MS), as reported previously [24]. The acquired mass spectral (MS) data, including peak picking, peak grouping, retention time correction, second peak grouping, and annotation of isotopes and adducts, were analyzed by XCMS software (Sciex 3.4.1). Raw data files were transferred into mzXML format, followed by the process using the XCMS, CAMERA, and MetaX toolbox using R software. A combination of the retention time (RT) and m/z data was used to identify each ion. A three-dimensional matrix, including arbitrarily assigned peak indices (RT-m/z pairs), sample names (observations), and ion intensity information (variables), was performed by recording the intensities of each peak. Kyoto Encyclopedia of Genes and Genomes (KEGG) and Human Metabolome Database (HMDB) were used for annotating the metabolites (only those with a mass difference between the observed and the database values <10 ppm were considered). Isotopic distribution measurements and an in-house fragment spectrum library of metabolites were used to validate the molecular formula of the metabolite. The intensity of peak data was preprocessed by MetaX [2], and features that were detected in <50% of quality control (QC) samples or <80% of biological samples were removed. The remaining peaks with missing values were imputed with the k-nearest neighbor algorithm to increase the data quality further.

### 4.10. Network Pharmacology

#### 4.10.1. Screening the Potential Active Ingredients and Prediction of AR-Related Targets

The active ingredients from each herb of SBL were obtained from Traditional Chinese Medicine Systems Pharmacology Database and Analysis Platform (TCMSP, https://tcmspw.com/tcmsp.php, accessed on 20 February 2024) using the keywords “Xanthii Fructus”, “Angelicae Dahuricae Radix”, “Saposhnikoviae Radix”, “Magnoliae Flos”, “Gentianae Radix et Rhizoma”, and “Verbena Herba”. The retrieved compounds were then screened by ADME parameters (oral bioavailability (OB) ≥ 30%, drug-likeness (DL) ≥ 0.18, blood–brain barrier (BBB) ≥ −0.3, and Caco-2 permeability > 0) and Lipinski’s rule (molecular weight (MW) ≤ 500, number of hydrogen bond acceptors (Hacc) ≤ 10, number of hydrogen bond donors (Hdon) ≤ 5, and octanol–water partition coefficient (LogP) ≤ 5) [25,26]. The related targets of different ingredients were obtained from TCMSP or predicted from the Swiss Target Prediction platform (http://www.swisstargetprediction.ch/, accessed on 20 February 2024.).

AR-related targets were obtained by searching “Allergic Rhinitis” and “Rhinitis, Allergic” in the GeneCards databases (https://www.genecards.org/, accessed on 20 February 2024), OMIM (https://www.omim.org/, accessed on 20 February 2024), DisGeNET (https://www.disgenet.org/, accessed on 20 February 2024), and Therapeutic Target Database (TTD, https://db.idrblab.org/ttd/, accessed on 20 February 2024) [11,26].

#### 4.10.2. SBL-AR-Related Target Screening and PPI Network Construction

The intersection of potential targets in SBL and AR-related disease targets was obtained by jvenn (https://jvenn.toulouse.inrae.fr/app/index.html, accessed on 20 February 2024). The intersection targets represented the potential targets of SBL for AR therapy. The protein–protein interaction (PPI) networks were constructed by the STRING database (http://string-db.org/, accessed on 28 February 2024) and removed the value under medium-confidence interactions with scores = 0.4. The visualization data were output from Cytoscape (version 3.10.2). The GO and KEGG enrichment analysis was performed by clusterProfiler and path view package; the obtained data were visualized by the bioinformatics web server (http://bioinformatics.com.cn/, accessed on 28 February 2024) [21].

#### 4.10.3. Molecular Docking

The molecular docking was performed by AutoDock Tools (version 1.5.6) and Autodock Vina software (version 1.1.2) to evaluate further the binding potential of the active ingredient of SBL to target protein in AR. The 3D structures of selected active components and target proteins were obtained from PubChem and PDB databases. The water molecules and ligands of protein and drug were removed by PyMol (version 2.4.0). AutoDockTools collected the binding energy of the selected drug–protein (version 1.5.6). The molecular docking results are visualized by PyMol (version 2.4.0) [26].

### 4.11. Statistical Analysis

All data are presented as mean + standard error of the mean (SEM), and Graph Pad Prism version 5 software (San Diego, CA, USA) was used to analyze the data of each group. The Wilcoxon rank-sum test (for two groups) and the Kruskal–Wallis test (for more than two groups) were used to compare differences among groups in microorganisms and metabolite studies. The relationship between microorganisms and metabolites was assessed using Spearman rank correlation analysis. For other comparisons of the two groups, results were analyzed using the unpaired Student’s *t*-test or nonparametric Mann–Whiney test (equal variances not assumed). Differences were considered statistically significant when the *p*-value < 0.05.

## 5. Conclusions

This is the first study to evaluate the therapeutic effect and mechanism of SBL decoction in HDM-induced AR by combining in vivo experiments, 16S rRNA sequencing, fecal metabolism, and a network pharmacology analysis. In vivo study verified that SBL could effectively alleviate allergic symptoms in the HDM-induced AR mice model by reducing the in situ inflammation in the nasal tissue, restoring the epithelial barrier, and suppressing the important inflammatory cytokine IL-6. Specifically, SBL can particularly rebuild the abundance and composition of gut microbiota and alter immune-related metabolism. We predicted that the SBL could regulate the key targets of IL-6 to exert an anti-inflammatory effect in AR via network pharmacology and further confirm in the HDM-induced AR mice model. In conclusion, SBL is an effective therapeutic approach for AR by gut microbiota and fecal metabolism to regulate the revealing of the IL-6 and Erk1/AKT/PI3K signaling pathways.

## Data Availability

The data presented in this study are available on request from the corresponding author.

## Supplementary Materials

The following supporting information can be downloaded at https://www.mdpi.com/article/10.3390/ijms25168655/s1.
