# Supplementary material for: Integrating 16S rRNA Sequencing, Microflora Metabolism, and Network Pharmacology to Investigate the Mechanism of SBL in Alleviating HDM-Induced Allergic Rhinitis
Source: Int J Mol Sci. 2024 Aug 8;25(16):8655. doi: 10.3390/ijms25168655 (PMC11354307; doi:10.3390/ijms25168655)
Supplement: Supplementary file 1 [file ijms-25-08655-s001.zip › Supplementary Figures.pdf]

Figure S1

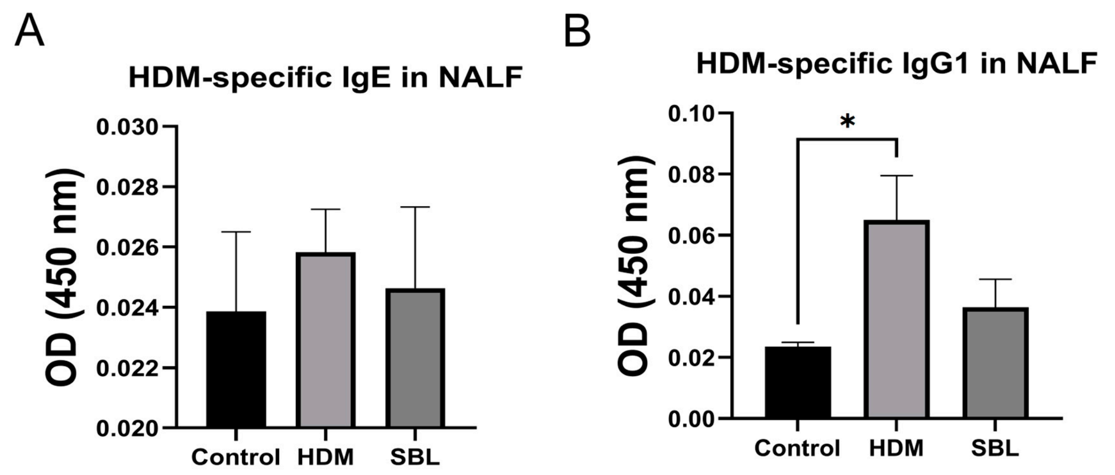

Figure S1. The effect of oral treatment of SBL on the levels of HDM-specific IgE and HDM-specific IgG1. HDM-specific IgE (A) and HDM-specific IgG1 (B) levels in the NALF were measured by ELISA. Results were presented with mean + SEM. $*p<0.05$. Control: healthy control; HDM: HDM-induced AR mice; SBL: SBL oral treatment on HDM-induced AR mice.

Figure S2

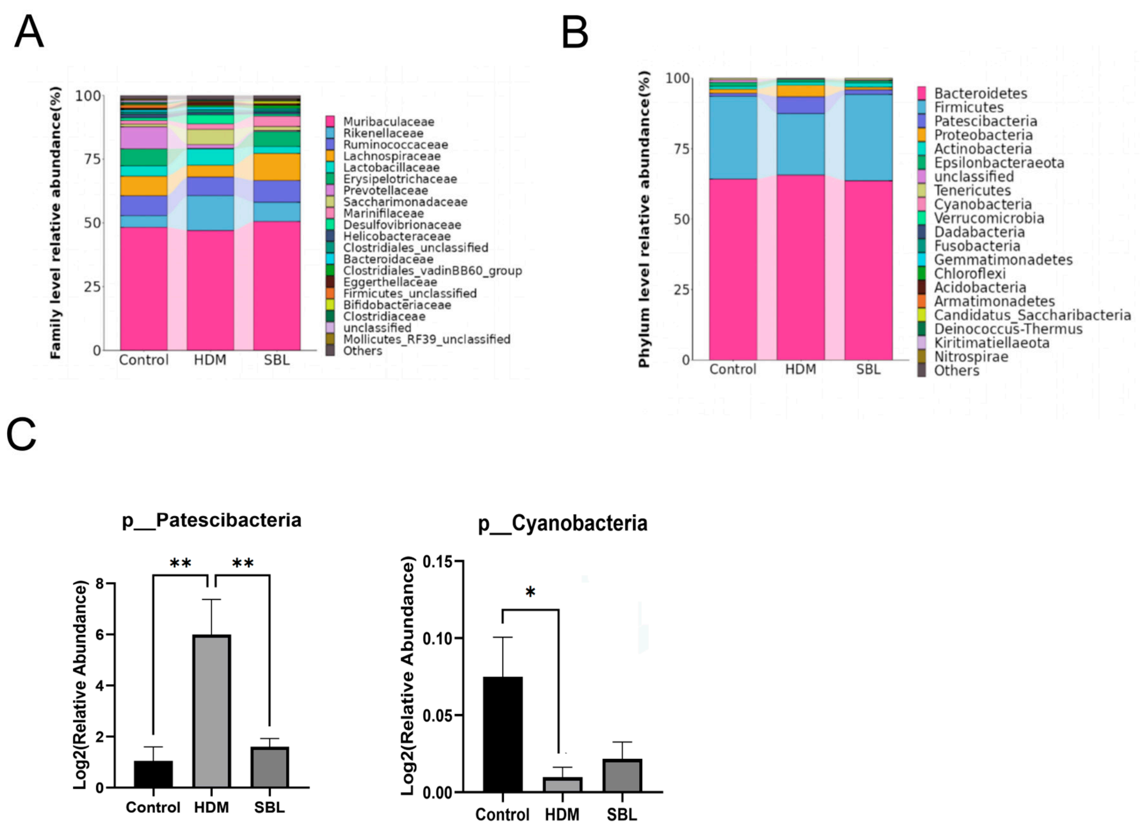

Figure S2. The abundance of differential bacteria in the three groups at the family and phylum level. Taxonomy classification of gut microbiota at the Family (A) and Phylum (B) levels among different groups was displayed in the top 20 enriched class categories. (C) Taxonomy classification of gut microbiota at the Phylum level among different groups displayed with the top 20 enriched class categories. The abundance of differential bacteria in the three groups at the phylum level was shown (C). Results were presented with mean + SEM. *p<0.05, **p<0.01, ***p<0.001. Control: healthy control; HDM: HDM-induced AR mice; SBL: SBL oral treatment on HDM-induced AR mice.

Figure S3

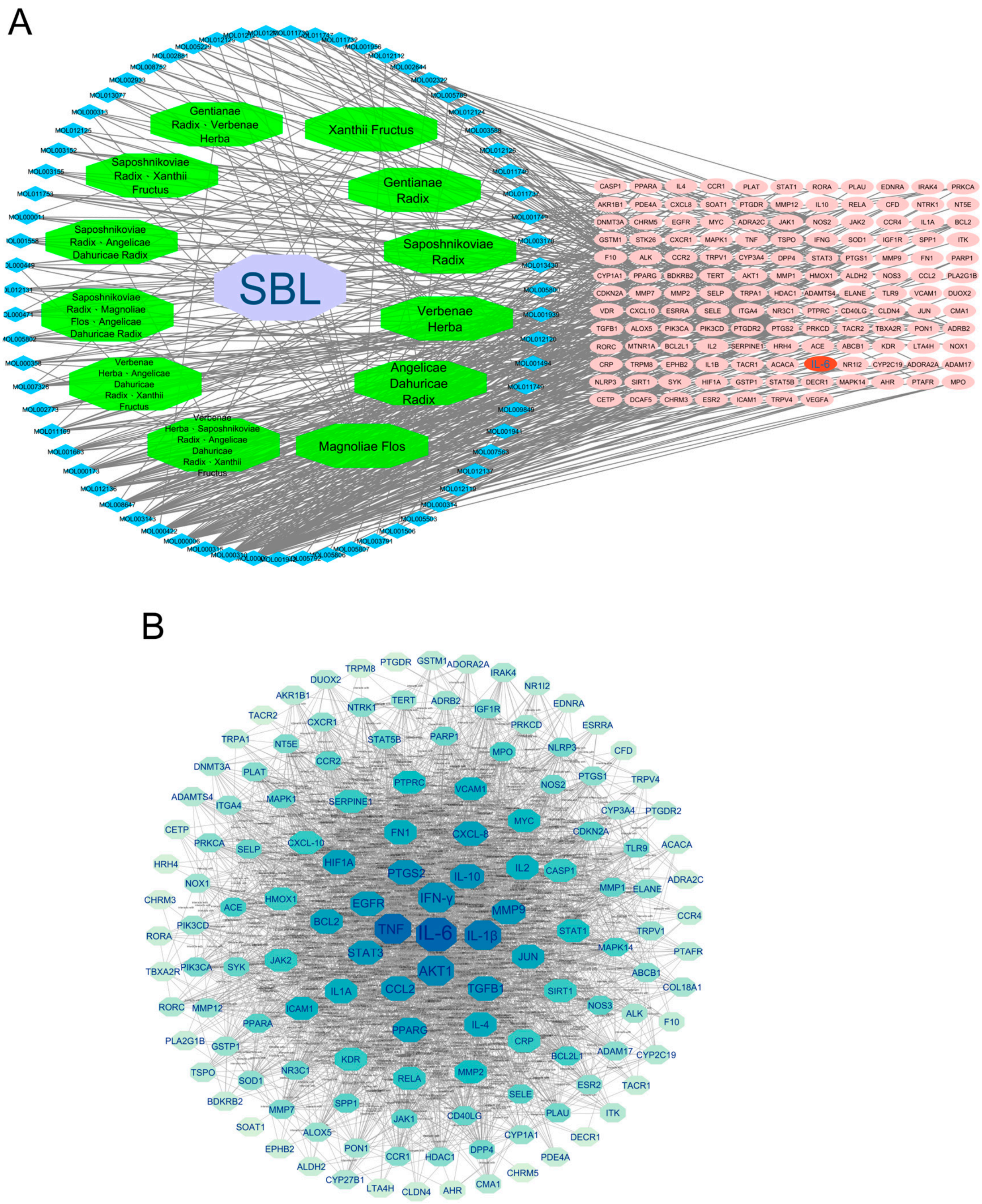

Figure S3. The pharmacological network of SBL and AR. (A) A network of active gradients and targets construction, the orange polygon represents traditional Chinese medicine, the blue diamond represents the active ingredient, and the pink oval represents the critical target. (B) In the topologic analysis of the PPI network, the 139 targets for the therapeutic effect of SBL in AR were obtained by degree; the nodes in different colors represent different degrees; blue color means high degree, and white color means low degree.
